# Adsorption of Pb^2+^and malachite green from water onto a newly developed nanocomposite of bentonite@perovskite Co-Ni oxide@bimetallic Mg/Cu MOFs and their adsorption and kinetic studies

**DOI:** 10.1038/s41598-026-42785-5

**Published:** 2026-04-27

**Authors:** Shymaa E. Adel, Ibrahim E. T. El Sayed, Elhassan A. Allam, Gehan M. Nabil, Mohamed E. Mahmoud

**Affiliations:** 1 Chemistry Department, Faculty of Science, Menoufia University, Shebin El-Koom, Menoufia Egypt; 2 Nuclear Power Plants Authority (NPPA), P.O. Box 11381, Cairo, Egypt; 3 Department of Chemistry, College of Science and Humanities in Al-Kharj, Prince Sattam Bin Abdulaziz University, Al-Kharj, 11942 Saudi Arabia; 4 Chemistry Department, Faculty of Science, Alexandria University, P.O. Box 426, Ibrahimia, Alexandria 21321 Egypt

**Keywords:** Adsorption, Pb^2+^, Malachite green, Bimetallic MOFs, Perovskite metal oxide, Nanocomposite, Bentonite, Chemistry, Environmental sciences, Materials science

## Abstract

In this study, an environmentally friendly nanocomposite was synthesized and comprehensively characterized for its potential to adsorb Pb^2+^ ions and malachite green dye (MG) from aqueous solutions. The composite material was based on nanobentonite intercalated with Ni–Co perovskite oxide and Bimetallic metal–organic frameworks (MOFs) from Bimetallic Mg/Cu, linked with tartaric acid as an organic linker. Structural and chemical properties of the individual components and the final composite were examined using FT-IR, XRD, BET, while TGA was employed to assess thermal stability. SEM analysis provided insights into the surface morphology. The adsorption performance was evaluated under varying pH levels, contact times, adsorbent dosages, and contaminant concentrations. The ultimate adsorption capacity for Pb^2+^ reached 105.7 mg/g at pH 7.0 with 10.0 mg of adsorbent and a contact time of 5.0 s. For MG, the highest uptake was 14.53 mg/g under optimal conditions of pH 6.0, 20.0 mg/L dye concentration, 20.0-minute contact time, and 10.0 mg of adsorbent.

**Supplementary Information:**

The online version contains supplementary material available at 10.1038/s41598-026-42785-5.

## Introduction

 Metal–organic frameworks (MOFs) are a class of porous crystalline structures constructed through the coordination of metal ions or clusters with organic linkers, creating highly ordered porous networks. Due to their tunable pore structures and large surface areas, MOFs and their composites have attracted growing research interest owing to their diverse applications in catalysis, water purification, drug delivery, and energy storage^1^. These hybrid structures are typically formed by combining mono-, di-, tri-, or tetravalent organic ligands with metal centers, resulting in diverse geometries and physicochemical properties^2^. Bimetallic MOFs, which incorporate two different metal ions within their framework, have attracted attention due to their enhanced tunability. Various strategies have been developed to design Bimetallic MOFs with well-defined compositions and structural stability especially Bimetallic based on Cu incorporated into other metals like Fe–Cu-based MOFs and Co–Cu MOFs^3–5^.

Perovskite oxides are considered among the most promising materials due to their unique chemical and physical properties, which have been widely exploited in applications such as energy storage, catalysis, and water remediation^6^. The name “perovskite” originates from Lev Perovski, a Russian scientist who first identified the mineral structure typical of this class of compounds. A well-known example is the ABO₃-type perovskite structure. In perovskite-type structures, the ‘A’ site is generally occupied by an alkaline earth metal, while the ‘B’ site contains a transition metal^7^. Various synthesis methods have been developed to prepare these nanoporous materials with diverse metal oxide compositions and enhanced functional characteristics by some methods as soft or hard template, hydrothermal, microwave, and combustion techniques^8^. In the context of water purification, Ni–Co-based perovskite oxides are particularly effective due to their large surface area, oxygen vacancies, and redox-active sites. These characteristics enhance their ability to adsorb heavy metal ions like Pb^2+^ through surface complexation and electrostatic interactions^9^.

Clays are effective adsorbents with various types such as bentonite, kaolinite, and sepiolite, each differing in structure and adsorption properties. Among them, bentonite, mainly composed of montmorillonite, stands out for its widespread availability, high surface area, chemical stability, low cost, and marked tendency toward metal ions and organic pollutants, these properties make it especially suitable for environmental remediation^10^. Recent studies have highlighted the enhanced sequestration heavy metals like Pb^2+^ and dyes such as MG using bentonite and its modified forms. Techniques like surface modification and nano structuring have further improved its adsorption performance under different environmental condition^11^. The use of naturally occurring clays such as bentonite and fluorapatite in the development of adsorbents has gained considerable attention due to their abundance, low cost, high surface area, and chemical stability. These materials serve as effective support matrices for functional polymers and composites, significantly enhancing adsorption capacities for environmental remediation of dye and heavy metal pollutants. Bentonite, in particular, provides excellent cation exchange properties and structural flexibility, making it suitable for surface modification and cross-linking with biopolymers like chitosan. Similarly, fluorapatite contributes additional active sites for improved pollutant interaction. Several studies have successfully utilized bentonite-based and fluorapatite-based composites in fabricating efficient adsorbents for organic dye removal, highlighting their potential in environmental remediation applications^12^. Pollution of aquatic environments with dyes and heavy metals remains a critical concern due to its significant impact on public health. Contaminated water can transmit a range of infectious diseases such as cholera, hepatitis, encephalitis, dysentery, and typhoid, posing serious risks to human health and the surrounding ecosystem^13^. Recent studies have demonstrated the effectiveness of advanced materials in tackling both environmental and biomedical challenges. For instance, multifunctional biopolymer-based films have shown significant potential in medical applications such as wound healing, owing to their inherent biocompatibility and antimicrobial properties. In the environmental sector, increasing attention has been given to the fate and behavior of pharmaceutical pollutants in agricultural systems, with research highlighting the need for integrated risk assessment approaches to understand the interactions within the water–soil–plant continuum. Moreover, the development of photocatalytic nanomaterials, such as Pd-doped ZnO, has opened new avenues for visible-light-induced degradation of dyes. persistent organic pollutants like antibiotics in water. These advancements collectively underscore the growing role of material science in addressing complex environmental and health-related problems^14^. Research has increasingly focused on nanomaterials, agricultural residues, and functionalized composites for the efficient removal of dyes, heavy metals, and pharmaceutical contaminants, demonstrating the potential of metal-doped semiconductors in advanced wastewater treatment. Similarly modified agricultural residues have been explored for the removal of synthetic dyes from wastewater, offering eco-friendly alternatives to conventional treatments. Other studies have developed composite adsorbents as kaolinite-cellulose/cobalt oxide hybrids, which exhibit strong adsorption capacities and tunable surface properties. Furthermore, advanced materials like metal–organic frameworks (MOFs) and hydrogel nanocomposites have shown great potential as candidates for hybrid water treatment systems, combining high surface area, selectivity, and reusability. Collectively, these studies emphasize the crucial role of innovative material design and adsorption mechanisms in advancing sustainable solutions for environmental remediation^15–17^. The sequestration of toxic metal species and contaminants from water can be achieved through various treatment methods; among them, has proven to be one of the most efficient methods because of its operational simplicity, low cost, and rapid processing time^18–20^.

Lead is one of the most dangerous heavy elements and it is a longstanding pollutant in water because of its serious effect if it is found in water without acceptable levels. The tolerable Pb^2+^ level in water is about 0.05 mgL^− 1^ in accordance with EPA^21^. Lead can adversely affect human health by causing brain damage, renal failure, and blood poisoning and it may hinder the oxygen-transporting efficiency of red blood cells throughout the body^22^. Several recent studies have focused on the creation of innovative adsorbent materials for efficient removal of lead from aqueous environments. These materials include nanocomposites, biochar-based systems, and functionalized frameworks, offering high surface area, selectivity, and reusability. Titanium oxide-bound α-aminophosphonate nanocomposites demonstrated strong affinity for both lead and copper ions, combining inorganic support with organic functional groups for improved adsorption performance^23^. Magnetic cellulose nanocomposite beads incorporating activated bentonite offered a reusable and efficient solution for lead removal from water^24^, while thiophene-functionalized metal–organic frameworks (MOFs) presented high selectivity and capacity due to targeted functionalization^25^. A MoO₃-biochar composite showed promising performance and provided mechanistic insights into lead adsorption processes^26^. Additionally, magnetic MOF composites exhibited dual functionality by simultaneously removing lead and MG from wastewater^27^. Finally, Fe–Mn/biochar composites stabilized with starch demonstrated effective lead elimination from both water and soil, emphasizing environmental applicability and stability^28^.

Malachite green (MG), a N-methylated diamino triphenylmethane dye, is an organic compound named for its characteristic green color. It is a cationic dye typically found in crystalline powder form and is known to decompose upon exposure to air and light^29^. MG is considered one of the most hazardous dyes and should be excluded from water resources due to its toxicological effects^30^. Its toxicity increases with prolonged exposure, higher temperature, and elevated concentrations. MG has been associated with liver tumors, a high risk of carcinogenic effects, chromosomal aberrations, and various histopathological impacts in humans^31–34^.

In this work, a novel and efficient ternary nanocomposite was synthesized for the first time via combination of intercalated nano-bentonite with perovskite Ni–Co oxide and bimetallic Mg/Cu-tartaric MOFs (Bent@Perov@BM-MOFs) for the aim of removal of Pb^2+^ ions and MG from water. This nanocomposite exhibited excellent adsorption capacity with rapid contaminant removal, highlighting its potential in practical wastewater treatment applications. The synthesis procedure was based on cost-effective methodology, utilizing readily available precursors such as bentonite, tartaric acid, and common metal salts (Mg, Cu, Ni, and Co chlorides) via simple protocol as co-precipitation and hydrothermal treatment that do not require specialized equipment or costly reagents^35–40^. In addition, the removal processes of Pb^2+^ and MG pollutants from aquatic system were conducted and accomplished in few seconds by using microwave assisted adsorption approach to add more dimension to the outlined method in this work.

## Experimental

###  Materials and Instrumentation

Tables 1 and 2 list the chemicals (AR grade, used as supplied) and instrumentation required in this study.

**Table 1:** The chemicals purity and its specifications.

Chemical name	MF	FW (g/mol)	Assay	Company
Magnesium chloride hexahydrate	MgCl_2_·6H_2_O	203.30	99.0%	Sigma Aldrich, USA
Copper chloride	CuCl_2_	134.45	99.0%	
Tartaric acid	C_4_H_6_O_6_	150.09	99.0%	
Nickel chloride hexahydrate	NiCl₂·6 H₂O	237.71	99.99%	
Cobalt chloride hexahydrate	CoCl_2_.6H_2_O	237.95	99.0%	
Sodium hydroxide	NaOH	40	99.0%	BDH, UK
Ethanol	CH_3_OH	46.07	99%	Elnasr chemicals, Egypt
Formalin	HCHO	30.03	37–40%	

**Table 2:** Instrumentations.

Instrument name	Model	Data	Conditions	Technique
Fourier-transform infrared spectro-photometer FT-IR	BRUKER Tensor 37	FT-IR spectrum	400–4000 cm^− 1^	Using KBr pellets
TGA-7 thermobalance	A Perkin-Elmer	Thermogram	Temperature heating (25–800 °C)	Pure nitrogen atmosphere, flow rate = 40 mL/min, heating rate = 10 °C/min and sample mass in the range of 5.0–6.0 mg
X-ray diffraction (XRD)	Shimadzu lab x 6100, Kyoto, Japan	XRD spectrum	40 kV, 30 mA, λ = 1 Å, 2θ from 10 to 80, recording steps of the diffraction data of 0.02°, at a time of 0.6 s, at room temperature (25 °C).	X-ray diffractometer, using target Cu-Kα
Scanning electron microscope SEM	JSM-lT200, JEOL Ltd	SEM images	Imaging mode	Sputtering coating (JEOL-JFC-1100E)

### Synthesis a composite of nano bentonite intercalated with perovskite Ni-Co oxide@bimetallic MOFs [Mg/Cu-T] nanocomposite

#### Combustion synthesis of Ni-Co nano perovskite oxide

Ni–Co perovskite oxide nanoparticles were synthesized through a combustion-assisted route. Initially, equimolar amounts (0.01 mol each) of nickel chloride hexahydrate (NiCl₂·6 H₂O) and cobalt chloride hexahydrate (CoCl₂·6 H₂O) were dissolved in 250 mL of deionized water under continuous stirring. Sodium hydroxide solution (0.04 mol in 50 mL deionized water) was then added dropwise to the mixed metal solution, and the reaction mixture was maintained at 80.0 °C with stirring for 90.0 min. The resulting greenish-brown Ni–Co hydroxides precipitate was collected, thoroughly washed with deionized water, and dried at 70.0 °C for 4.0 h. To obtain the perovskite oxide phase, 3.0 g of the dried Ni–Co hydroxides precursor was homogeneously mixed with 3.0 g of urea and thermally treated at 700.0 °C for 4.0 h in a muffle furnace. After combustion, black Ni₀.₅Co₀.₅O nanoparticles were obtained^10^.

#### Synthesis of bimetallic Mg/Cu-tartaric MOFs

The Mg/Cu-tartaric acid bimetallic MOFs were prepared via a co-precipitation method. Copper chloride dihydrate (3.36 g) and magnesium chloride hexahydrate (5.08 g) were dissolved in 50 mL of deionized water to form a homogeneous solution. Separately, Tartaric acid (9.98 g) and sodium hydroxide (4.0 g) were dissolved in 100 mL of deionized water. The ligand solution was gradually added to the metal solution under continuous stirring at 80.0 °C for 90.0 min. A greenish-white precipitate was formed, which was subsequently filtered, washed several times with deionized water, and dried at 50 °C for 7.0 h to yield the Mg/Cu-tartaric MOFs^11^.

#### Synthesis of nano bentonite intercalated with perovskite Ni-Co oxide@bimetallic MOFs [Mg/Cu-T] nanocomposite

In the final fabrication step, equal amounts (2.50 g each) of nano-bentonite, Ni–Co perovskite oxide, and bimetallic Mg/Cu-tartaric MOFs were dispersed in a 250.0 mL of mixed solvents system consisting of distilled water, and formaldehyde with a volume ratio of 4:1. The suspension was refluxed under continuous stirring for 7.0 h to ensure effective intercalation and homogeneous integration of all components. After completion of the reaction, the obtained solid was repeatedly washed with deionized water to remove any unreacted species and residual solvent, followed by drying to yield the Bent@Perov@BM-MOFs nanocomposite.

### Adsorption Pb^2+^ and MG from water onto Bent@Perov@BM-MOFs nanocomposite

The structural, chemical, and morphological properties of the synthesized Bent@Perov@BM-MOFs nanocomposite were characterized using Fourier-transform infrared spectroscopy (FT-IR), X-ray diffraction (XRD), thermogravimetric analysis (TGA), and scanning electron microscopy (SEM). Adsorption experiments were conducted to determine the optimal removal conditions of Pb^2+^ and MG. Microwave -assisted adsorption was employed for Pb^2+^, while a shaker-based method was used for MG. Key parameters investigated included solution pH, contact time, adsorbent dosage, and initial contaminant concentration.

### Adsorption experiments

A Standard calibration curve was prepared for MG. On the other hand, the Pb^2+^ concentration was determined by EDTA complexometric titration using xylenol orange as a visual indicator. The titration was performed under buffered conditions, with the pH adjusted to approximately 5.5 using a hexamine buffer to ensure selective complexation. The volume of EDTA consumed was used to calculate Pb^2+^ concentrations. The concentration of MG in aqueous solution was detected using a UV–visible spectrophotometer. The absorbance measurements were carried out at a maximum wavelength (λmax = 617 nm), corresponding to the characteristic absorption peak of MG in water in the range of 10–100 mg/L, exhibiting good linearity. Deionized water was used as a blank for all measurements to get rid of the interference. Each sample was analyzed in triplicate to achieve consistent and accurate results. This method enables accurate quantification of MG in aqueous solutions and is widely used to evaluate adsorption efficiency under varying experimental conditions such as pH, adsorbent dose, and contact time.

#### Optimization of pH

To investigate the effect of solution pH on the adsorption of Pb^2+^ and MG onto Bent@Perov@BM-MOFs, 10.0 mg of the nanocomposite was dispersed in 9.0 mL of buffer solutions with pH values ranging from 1.0 to 7.0 for Pb^2+^ and from 1.0 to 10.0 for MG. Subsequently, 1.0 mL of 0.1 mol/L Pb^2+^ solution or 2.0 mL of MG solution (20.0 mg/L) was added. Pb^2+^ adsorption experiments were conducted under microwave irradiation (800 Watts) for 15.0 s, while MG adsorption was performed by shaking the mixtures for 10.0 min at room temperature. After adsorption, the nanocomposite was separated by filtration. The residual Pb^2+^ concentration was determined by EDTA complexometric titration using hexamine buffer and xylenol orange indicator, whereas the remaining MG concentration was measured by UV–Vis spectrophotometry. The adsorption capacity was calculated using Eq. (1).

1 $${\text{Q }}={\text{ }}\left( {{{\mathrm{C}}_{\mathrm{o}}} - {\mathrm{C}}} \right){\text{ V}}/{\mathrm{m}}.{\text{ AW of adsorbate}}$$

#### Contact time optimization

The effect of contact time on the adsorption behavior of Pb^2+^ and MG was investigated to evaluate the adsorption kinetics. Experiments were conducted using 10.0 mg of the nanocomposite dispersed in 9.0 mL of buffer solution at the optimal pH. For Pb^2+^ adsorption, 1.0 mL of 0.1 mol/L Pb^2+^ solution was added and the mixture was exposed to microwave irradiation for contact times ranging from 1.0 to 40.0 s. For MG adsorption, 10.0 mL of dye solution prepared in the optimal buffer was mixed with the nanocomposite and shaken for contact times between 1.0 and 30.0 min. After adsorption, the same procedure described in the pH optimization experiments was followed, and the adsorption capacity was calculated using Eq. (1).

#### Nanocomposite dosage

The influence of nanocomposite dosage on the removal efficiency of Pb^2+^ and Malachite Green (MG) was examined while maintaining all other experimental parameters at their optimized values. Various amounts of the nanocomposite from 10.0 to 100.0 mg were introduced into buffer solutions adjusted to the optimal pH. In the case of Pb^2+^ adsorption, 1.0 mL of a 0.1 mol/L Pb^2+^ solution was added, and the system was subsequently treated under microwave irradiation for the predetermined equilibrium time. For MG adsorption, different dosages of the nanocomposite were added to 10.0 mL of dye solution prepared in the optimal buffer, followed by agitation for the optimized contact time. Upon completion of the adsorption process, the remaining concentrations of Pb^2+^ and MG were analyzed according to the procedure described previously, and the adsorption capacity was calculated using Eq. (1)

#### Initial concentration effect

The influence of initial Pb^2+^ and MG concentrations on adsorption was examined under optimized conditions. 10 mg of Nanocomposite was added to solutions of varying concentrations (Pb^2+^: 0.2–2 mol/L, MG: 10–100 mg/L). Pb^2+^ samples were treated with microwave irradiation, while MG samples were shaken for the optimized time. After separation, residual concentrations were measured using EDTA titration (Pb^2+^) and UV–Vis spectrophotometry at 617 nm (MG). The adsorption capacity was calculated using Eq. (1).

## Results and discussion

### Characterization of Bent@Perov@BM-MOFs nanocomposite

**Scheme 1:**
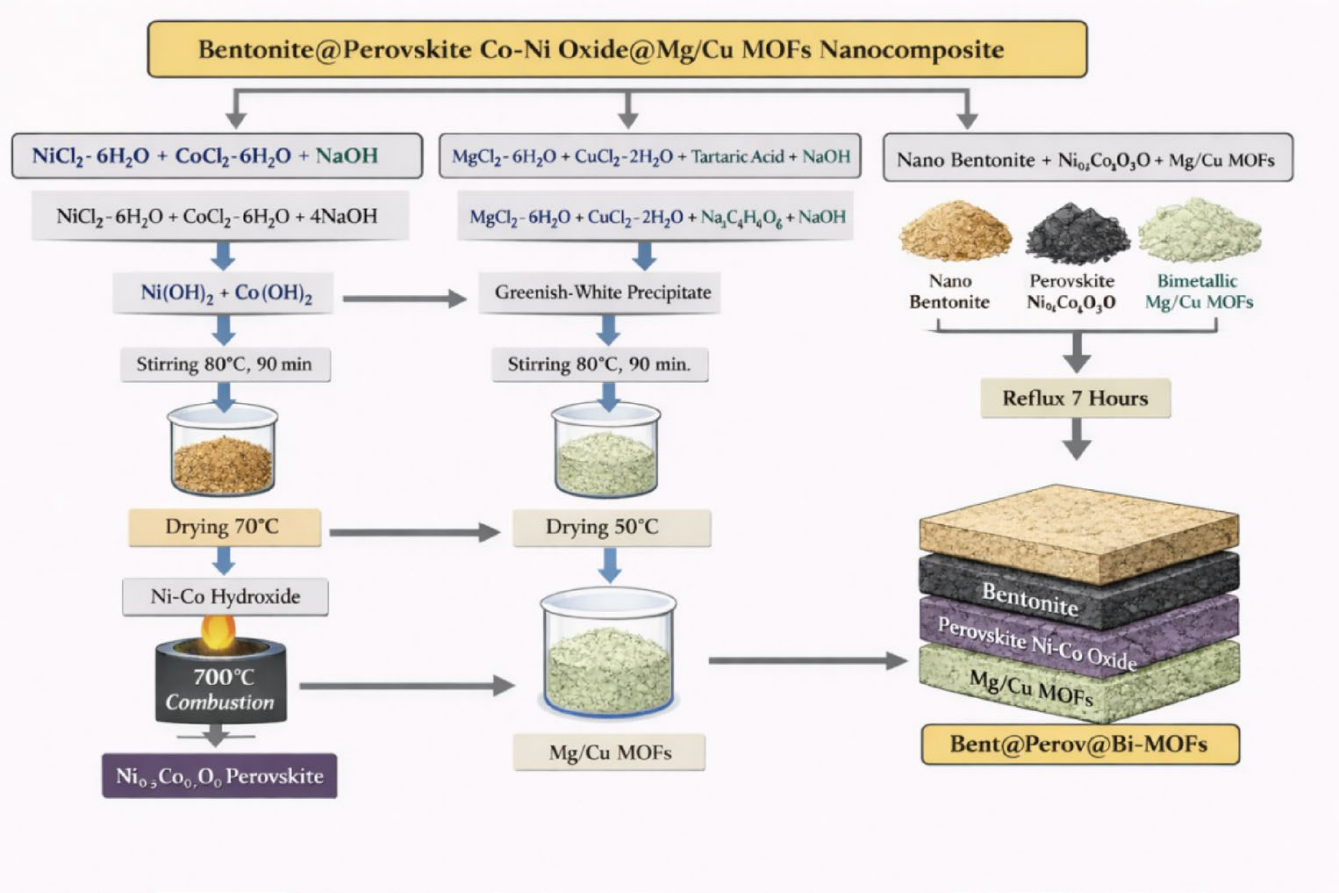
Different synthesis steps of the aimed Bent@Perov@BM-MOFs nanocomposite.

In this work, the Bent@Perov@BM-MOFs nanocomposite was fabricated through a formaldehyde-assisted reflux process that promotes strong interfacial integration among all components. The structural stability of the composite is ensured by a synergistic network of interactions, including hydrogen bonding between bentonite surface hydroxyl groups (≡ Si–OH/≡Al–OH) and the carboxylate or hydroxyl functionalities of the Mg/Cu–tartaric MOFs, electrostatic attractions between negatively charged bentonite layers and exposed Ni^2+^/Co³⁺ sites of the perovskite oxide, and coordination bonding between MOF carboxylate oxygen atoms and surface metal centers of the Ni–Co perovskite. Additionally, metal–oxygen–carbon (M–O–C) linkages and hydroxyl-bridged interactions (M–O–Si and M–OH–M) further anchor the perovskite phase to the bentonite matrix. The presence of formaldehyde under reflux conditions facilitated the formation of methylene (–CH₂–O–) bridges between adjacent hydroxyl groups, converting weak physical contacts into semi-covalent cross-linked networks. Collectively, these interactions generated a chemically immobilized, multi-bonded architecture that stabilized and preserved the structural integrity of the nanocomposite and effectively suppresses the leaching of individual components during the adsorption process. Finally, Scheme 1 represents the synthesis steps of the aimed nanocomposite.

#### FT-IR analysis

**Fig. 1:**
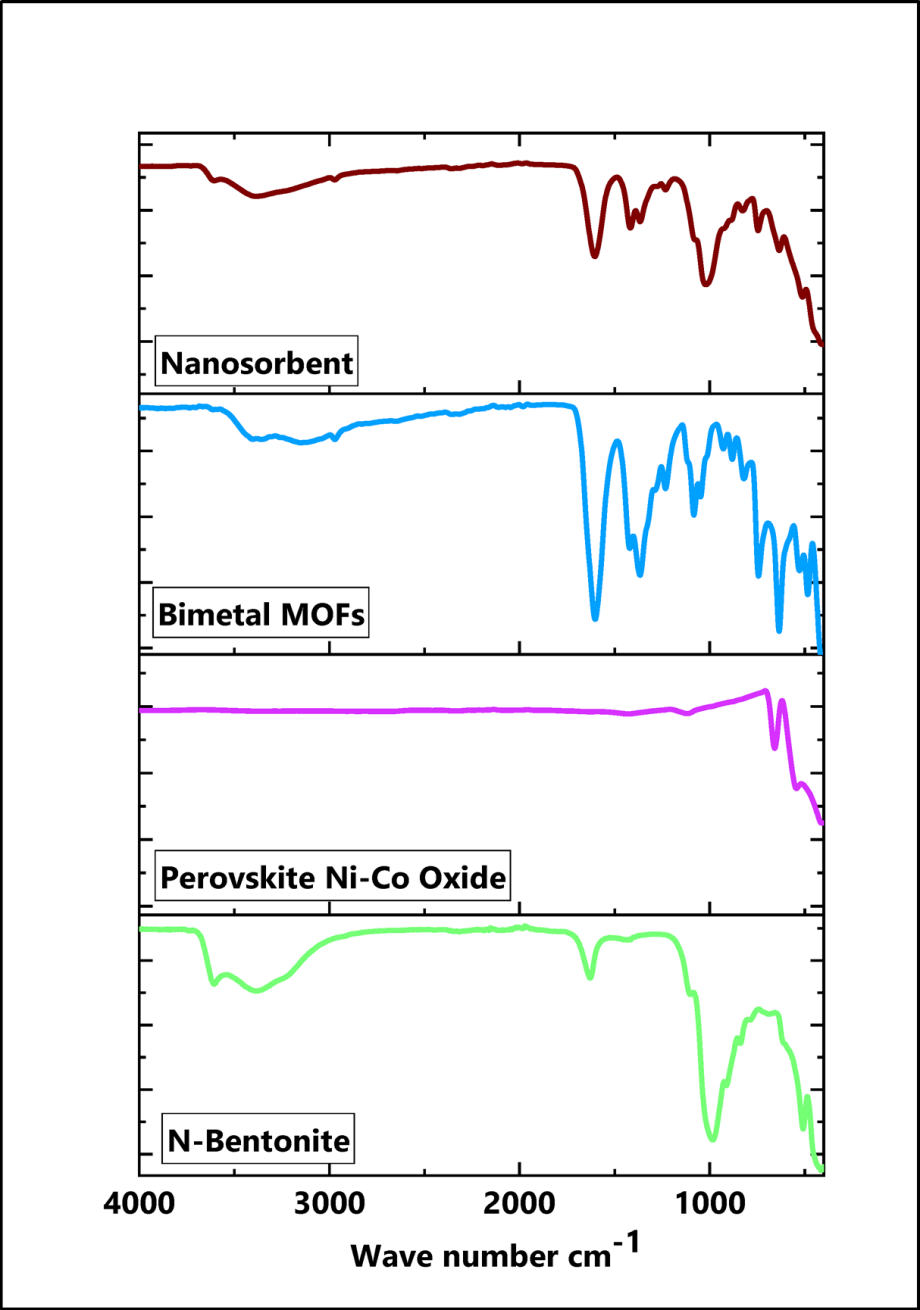
FT-IR spectra of Nano bentonite, perovskite Ni-Co Oxide, Bimetallic MOFs, and Bent@Perov@BM-MOFs nanocomposite.

FT-IR spectroscopy (500–4000 cm^−1^) was used to investigate the nanocomposite and its components, as illustrated in Fig. 1. Nanobentonite exhibited characteristic peaks at 509.25 cm^−1^ (Al–O–Si octahedral vibrations and Si–O–Si bending), 787.62 and 838.92 cm^−1^ (out-of-plane Al–O and Si–O vibrations), and 1102.55 cm^−1^ (Si–O stretching in the clay plane)^9^. Broad bands at 1629.83 and 3383.45 cm^−1^ were attributed to OH bending and stretching of adsorbed water, while the peak at 3605.88 cm^−1^ corresponds to hydroxyl-coordinated Al³⁺ ions. The peaks at 543.98, 658.17, and 1118.33 cm^−1^ indicate the presence of Ni–Co perovskite oxide. Several absorption bands detected below 1000 cm^−1^ in the FT-IR spectrum of the Mg/Cu-T bimetallic MOFs are related to metal-oxygen vibrational modes. These signals, appearing at 527.63, 635.29, 743.09, 820.44, 828.42, and 881.79 cm^−1^, arise from the Mg–O and Cu–O bonds that constitute the inorganic framework of the material^36^. The peak at 1083.18 cm^− 1^ is owing to C-OH bonds. The efficient deprotonation of carboxyl and the coordination of the metals Cu and Mg in the newly created metal organic frameworks are shown by the absence of the typical carboxyl signal at 1712.0 cm^− 1^^36^. The peaks from 1000 to 1400 cm^− 1^, 1047.84, 1083.18, 1232.98, 1288.47, and 1367.11 are due to the tartaric acid skeleton’s C-H bending vibrations besides, while the peak at 1420.55 cm^− 1^ is noted to the carboxylate groups (CO_2_) vibrational spectrum and O-H deformation in tartaric acid carboxylate groups. The 1602.45 cm^− 1^ peak is generated by the tartaric acid COOH and C = O stretching vibration. The 2971.65 cm^− 1^ peak is distinctive of C–H stretching, and 3357.24 cm^− 1^ peak is ascribed for the tartaric acid hydroxy groups owing to O–H stretching^36^. A slight shift in the Si–O–Si stretching vibration band from 1102 cm^−1^ in the native bentonite to 1098 cm^−1^ in the nanocomposite. This suggests structural interactions between the clay and incorporated phases. Additionally, a new absorption band around 570 cm^−1^ is attributed to metal–oxygen (Co–O) vibrations, indicating successful integration of the Co–Ni perovskite component into the hybrid material^35,38^.

#### X-ray diffraction

**Fig. 2:**
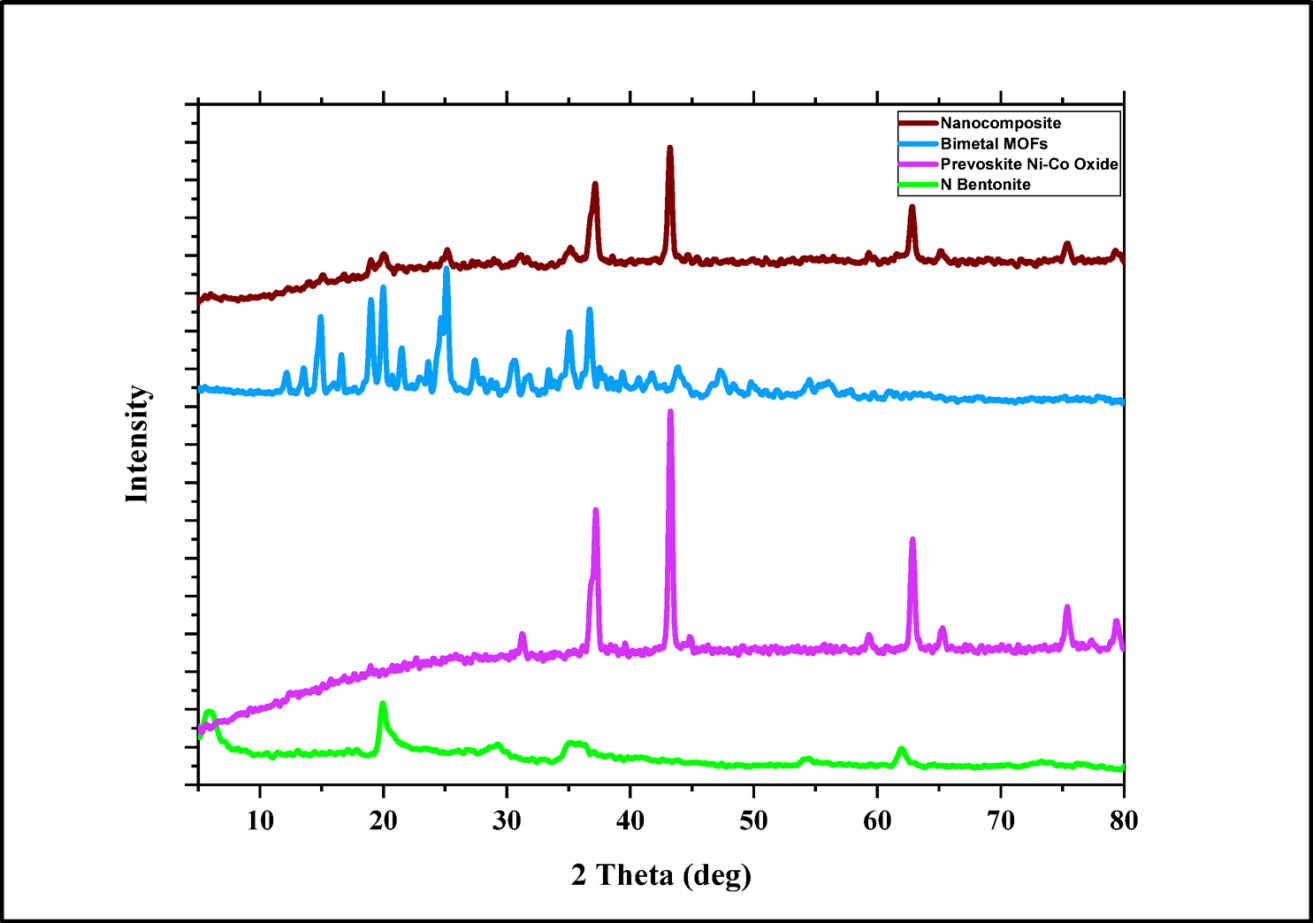
XRD spectra of nano bentonite, perovskite Ni-Co Oxide, Bimetallic MOFs, and Bent@Perov@BM-MOFs nanocomposite.

**Fig. 3:**
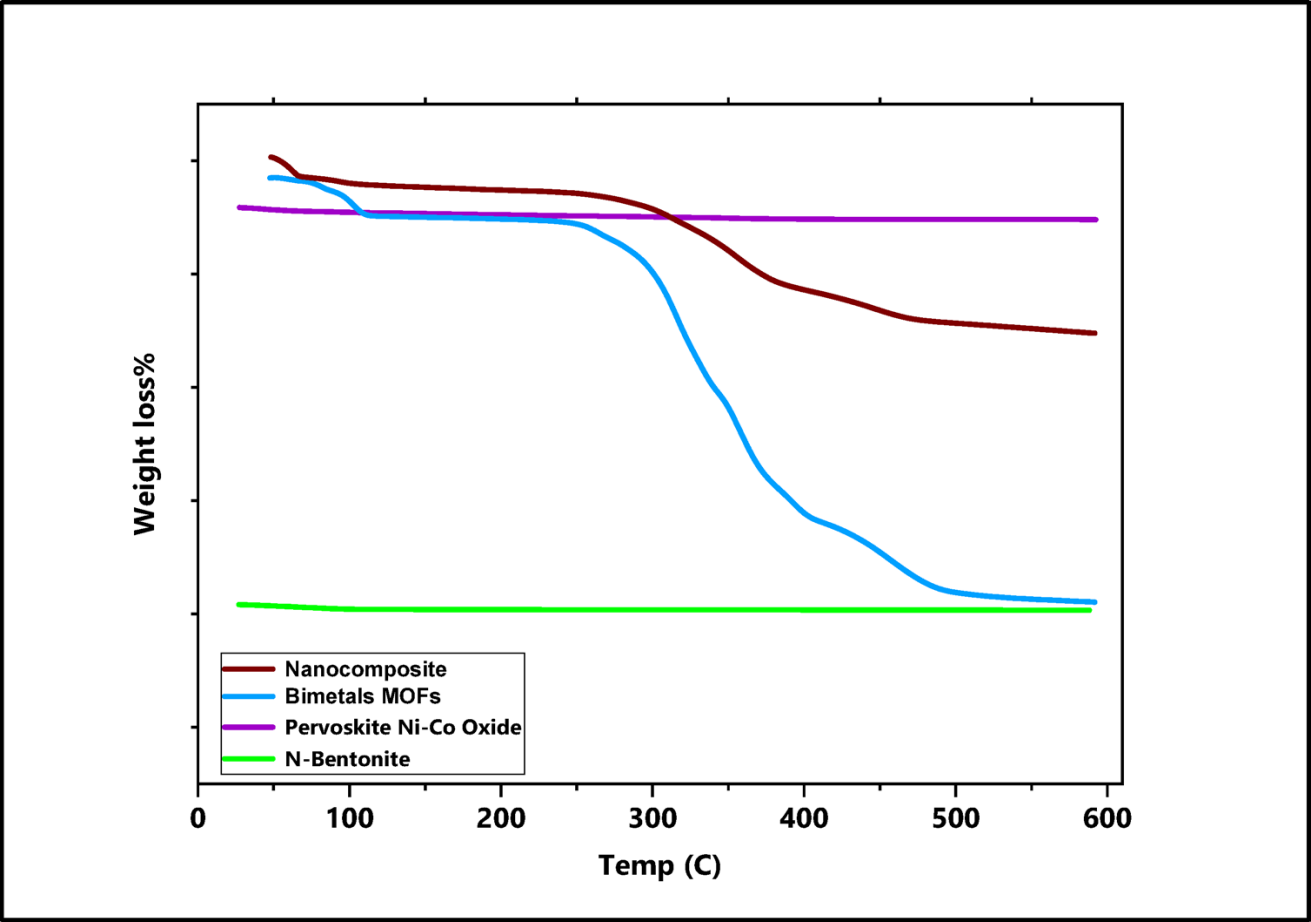
TGA of nano bentonite, perovskite Ni-Co oxide, bimetallic MOFs, and Bent@Perov@BM-MOFs nanocomposite.

 XRD analysis was carried out to investigate the crystalline nature of the Bent@Perov@BM-MOFs nanocomposite and verify its successful formation as represented in Fig. 2. The resulting diffraction pattern confirmed the presence of nanobentonite, Ni–Co perovskite oxide, and Bimetallic Mg/Cu MOFs in the composite. As shown in Fig. 3, distinct peaks appeared at 2θ = 17.96°, 21.38°, 24.40°, 25.21°, 25.82°, 27.93°, 33.37°, 36.37°, 37.20°, 38.67°, 43.23°, 59.36°, 62.79°, 65.24°, and 75.36°, which correspond to the crystalline phases of the composite components. All diffraction peaks were carefully assigned by comparison with standard JCPDS reference patterns as previously reported in the literature data for bentonite, Ni–Co mixed oxides, and Mg/Cu-based MOF as listed in Table 3^9^. Overlapping peaks were carefully considered, and only well-resolved reflections were used for phase identification. Prominent peaks at 2θ = 26.5°, 36.8°, and 43.1° correspond to the (220), (311), and (400) planes of spinel-type Co–Ni oxide, consistent with standard patterns (e.g., JCPDS No. 00–042-1467 for Co₃O₄). Peaks around 6.9° and 19.8° confirm the presence of layered bentonite, in accordance with^10,36^. Additional reflections near 11.7° and 16.3° are attributed to the crystalline features of MOFs derived from Mg/Cu–tartaric acid frameworks, consistent with reported literature. The absence of unassigned peaks suggests successful integration of all components without significant impurity formation. Notably, the (001) diffraction peak of bentonite shifted from 5.7° to 5.4° 2θ in the composite, indicating interlayer expansion due to intercalation. Minor peaks at 29.5° and 32.1° may also correspond to Co–Ni mixed oxide phases such as Co₀.₁Ni₀.₉O (JCPDS 04–018-7323)^8,9^, supporting the successful incorporation of Co and Ni into a unified oxide structure (Tables 3, 4).

**Table 3:** XRD pattern list of the Ni–Co perovskite oxide according to XRD reference codes.

Ref. code	Chemical formula	Compound name
00–001-1152	CoCo_2_O_4_	Cobalt oxide
04–018-7323	Co_0.1_Ni_0.9_ O	Cobalt nickel oxide
00–042-1467	Co_3_O_4_	Cobalt oxide
04–006-1775	Co_0.5_Ni_0.5_O	Cobalt nickel oxide

**Table 4:** Kinetic parameters for the adsorption of Pb^2+^ and MG onto Bent@Perov@BM-MOFs.

Kinetic models	Equations	Kinetic parameters	Pb^+ 2^	Malachite green
Pseudo-First Order	Ln (q_e_−q_t_) = Ln (q_e_) − k_1_t	q_e_(mg g^− 1^)	31.12	11.46
		K_1_ (min^− 1^)	0.003	−0.004
		R^2^	0.943	0.972
Pseudo-Second Order	t/q_t_= 1/k_2_q_e_^2^+ t/q_e_	q_e_(mg g^− 1^)	43.47	14.49
		K_2_ (g mg^−1^min^− 1^)	145.52	32.03
		R^2^	0.772	0.992
Intraparticle Diffusion	q_t_ = k_id_ t^1/2^ + C	K_id_ (mg g^−1^min^− 1/2^)	1.25	0.78
		C	−4.11	−0.90
		R^2^	0.944	0.902
Elovich	q_t_ = 1/β Ln(αβ) + 1/β Ln (t)	α (mg g^−1^min^− 1^)	0.24	0.41
		β (mg g^− 1^)	0.39	0.38
		R^2^	0.887	0.866

#### Surface area and porosity

To investigate the textural properties of the synthesized nanocomposite, nitrogen adsorption–desorption measurements were performed. The resulting isotherm indicated the presence of a mesoporous structure, which facilitates efficient diffusion and greater accessibility for adsorbates. The Brunauer–Emmett–Teller (BET) surface area was calculated to be 152.4 m²/g, with a pore volume of 0.32 cm³/g and an average pore diameter of 8.4 nm as illustrated in Fig. 1S (Supplementary material). These structural characteristics suggest that the composite offers a highly accessible surface and an interconnected porous network, both of which are advantageous for the adsorption of heavy metal ions and dye molecules from aqueous media^40–44^.

#### Thermal gravimetric analysis

Using TGA equipment, the thermal stability of the produced nanocomposite and its authentic components were looked into and evaluated as shown in Fig. 3. Two stages of thermal degradation were observed in the thermogram of nanobentonite clay. The first stage, occurred between 19.35 and 104.35 °C and corresponds to the loss of physically adsorbed water, resulting in a weight loss of approximately 15.43%. The weight loss in the second stage, which transpired between 104.35 and 692.03 °C, was roughly 5.70%  since the nano bentonite clay’s ash content had decreased. Generally, the nanobentonite has excellent thermal stability without losing weight^10^. Three phases of thermal degradation are demonstrated in the thermogram curve of Bimetallic Mg-Cu MOFs. The first stage occurred approximately 17.58 and 126.39 °C. Due to some water on the MOF’s surface, there was a 0.60% weight loss. The second step took place at 126.39–341.13 °C. About 2.70% of the weight was shed due to tartaric acid’s two hydroxy groups breaking down. About 60.65% of the weight had been eliminated at 341.13–599.92 °C in the third stage. This stage was brought on by the tartaric acid’s skeleton breaking and the absence of its various carbon, hydrogen, and oxygen components. Because two types of metal oxides, MgO and CuO, were found in the finished items based on computation of molecular mass from the beginning up until the gravimetric measurement, the residual of the MOFs was approximately 36.05%, and this is a sign that the MOFs synthesis technique was developed^36^. The thermogram of the Bent@Perov@BM-MOFs nanocomposite provides proof of the effectiveness of the nanocomposite the overall weight loss of 46.77% throughout the production process proves that the mixing procedure between Nano-bentonite, Perovskite Ni-Co oxide, and Bimetallic MOFs transpired in the subsequent percentage: 1:1:1 which was clarified in the section of preparation^36^.

#### Scanning electron microscopy (SEM)

**Fig. 4:**
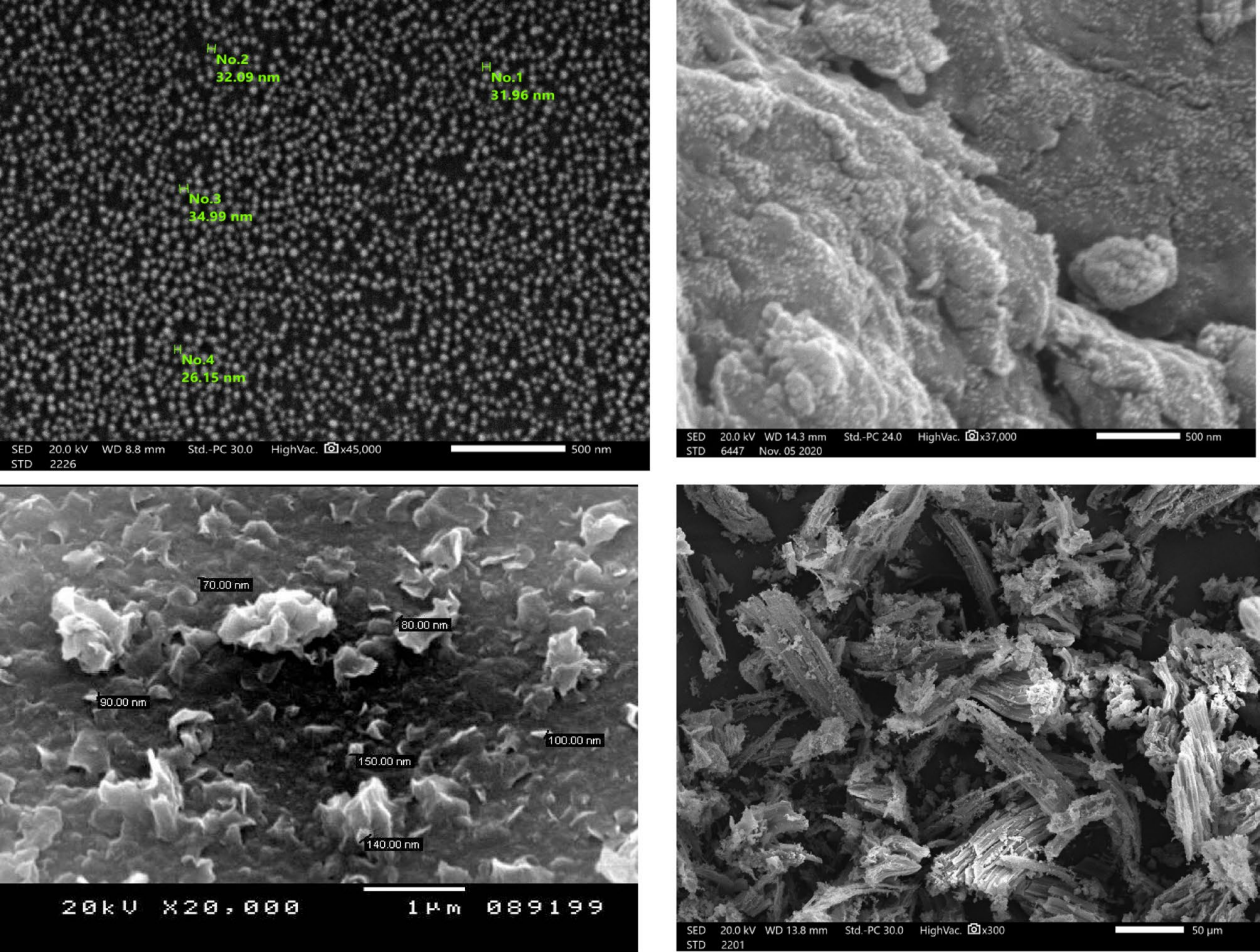
SEM image of nano bentonite, perovskite Ni-Co Oxide, Bimetallic MOFs, and Bent@Perov@BM-MOFs nanocomposite.

Nano bentonite, perovskite, Bimetallic MOFs [Mg/Cu-T], and Bent@Perov@BM-MOFs nanocomposite surface morphology were characterized by using SEM as explicated in Fig. 4a, b, c, and d, in that order. The SEM image of nano bentonite was depicted on Fig. 4a that appears as sheets of nanobentonite; Fig. 4b illustrates MOFs as a spherical form because of the Bimetallic MOFs’ uniform structure. Figure 4c exhibits heterogeneous structure because of the existence of nano perovskite Ni-Co oxide; and finally, Fig. 4d indicates the intercalation of nanobentonite with perovskite Ni-Co oxide, and the Bimetallic MOFs. The surface of Bent@Perov@BM-MOFs nanocomposite via SEM appeared to be a non-homogenous flower with Points of non-homogeneous rock above it, as demonstrated in Fig. 4d^32,36^. The updated SEM images were acquired at higher magnification (20,000×) and are now presented with calibrated scale bars in Fig. 4. These enhanced images provide better resolution and reveal a distinctive flower-like structure composed of layered, petal-like aggregates. The non-uniform arrangement suggests a hierarchical and porous surface architecture, which is favorable for adsorption by increasing the available surface area and providing varied active sites. This visual evidence supports the structural heterogeneity of the synthesized nanocomposite.

### Adsorption of Pb^2+^ and MG onto Bent@Perov@BM-MOFs nanocomposite

#### The pH effect on the adsorptive removal

**Fig. 5:**
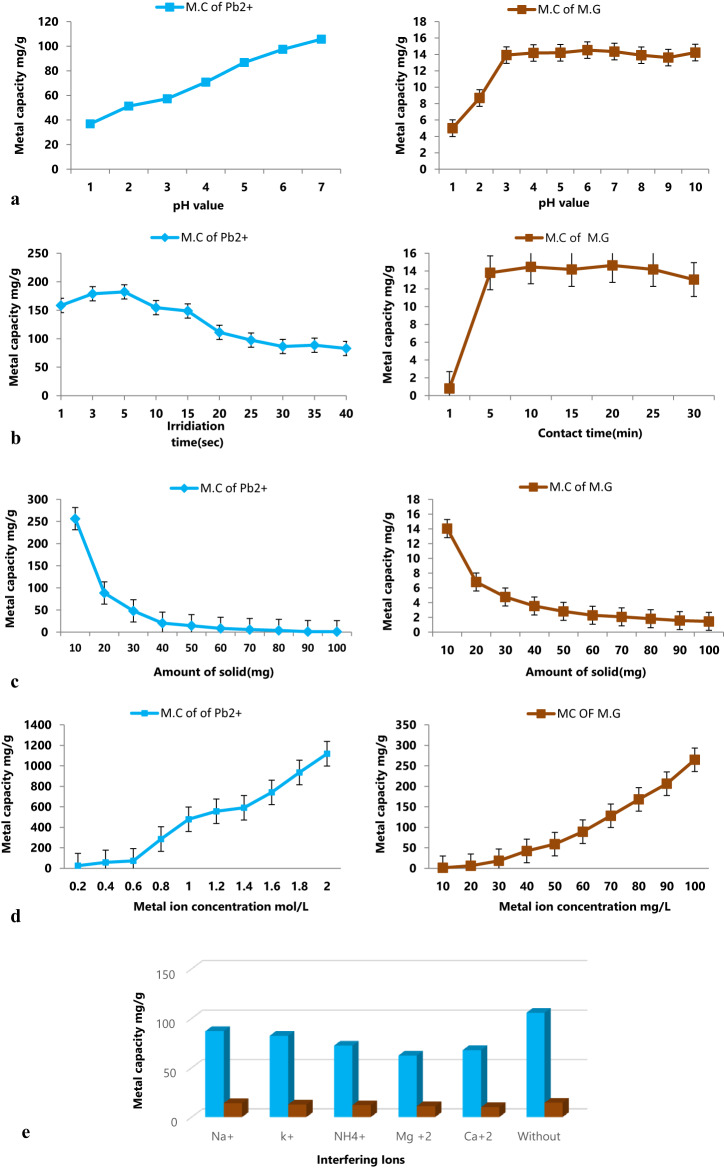
(**a**) Adsorption of Pb^2+^ and Malachite Green onto Bent@Perov@BM-MOFs nanocomposite at different pH values. (**b**) Adsorption of Pb^2+^ and Malachite Green onto Bent@Perov@BM-MOFs nanocomposite at different contact time. (**c**) Adsorption of Pb^2+^ and Malachite Green onto Bent@Perov@BM-MOFs nanocomposite at different amount of solid. (**d**) Adsorption of Pb^2+^ and Malachite Green onto Bent@Perov@BM-MOFs nanocomposite at different initial concentrations. (**e**) Adsorption of Pb^2+^ and Malachite Green onto Bent@Perov@BM-MOFs nanocomposite at different interfering ions.

Studying the impact of divergent pH values on the adsorption process was investigated in the experiments on adsorption using pH readings (1.0–7.0), and (1.0–10.0) for the Pb^2+^, and MG respectively for determination the maximum pH for the adsorption process for each of them. The consequences are displayed in Fig. 5a, hence the adsorption effectiveness of Pb^2+^ and MG were displayed in relation to numerous values of pH (1.0–7.0) for Pb^2+^, and pH (1.0–10.0) for MG. Several functional groups in this nanocomposite, such as the hydroxyl functional groups in bentonite, can aid in the process of adsorption, Moreover the perovskite Co-Ni oxide that comprises functional groups that are mostly derived from hydroxide groups, and those two materials were intercalated with and the mesoporous structure of Bimetallic MOFs. Figure 5a indicates that for Pb^2+^ the elimination efficiency in the minimal pH range of pH 1.0, 2.0, and 3.0 was 36.81, 51.28, and 57.26 mg/g respectively, and 105.71 mg/g was the most elevated adsorption attained at pH 7.0. Conversely, for MG, the lowest removal capacity was 5.0 mg/g at pH 1.0, while the maximum adsorption capacity (14.53 mg/g) was achieved at pH 6.0. This behavior can be attributed to the cationic nature of both Pb^2+^ ions and Malachite Green molecules. MG is a positively charged dye and is well known for its severe toxicological effects on aquatic organisms and human health^41^. At low pH values, the high concentration of H⁺ ions competes with Pb^2+^ ions and MG cations for the available active adsorption sites, leading to a significant decrease in adsorption efficiency. In contrast, as the pH increases, the concentration of hydrogen ions decreases, reducing this competition. Consequently, more cationic pollutants can interact with the negatively charged surface of the nanocomposite, leading to higher adsorption capacity^36^.

#### Kinetic of the adsorption removal at different contact times

Adsorption of Pb^2+^ and MG has been generated at divergent time intervals (1.0, 5.0, 10.0, 15.0, 20.0, 25.0, 30.0, 35.0, and 40.0 s) and (1.0, 5.0, 10.0, 15.0, 20.0, 25.0 and 30.0 min) utilizing a batch approach by using the microwave assisted method for Pb^2+^and the shaker for MG to make a contact between the contaminants in the aqueous solutions and the nanocomposite. For Pb^2+^, the removal efficiency increased sharply within the first 5.0 s, with adsorption capacities of 158.36, 178.80, and a maximum of 182.21 mg/g at 5.0 s. However, a gradual decline was observed from 10.0 to 40.0 s, where the capacity decreased from 154.53 to 82.88 mg/g. This non-typical trend may result from microwave-induced surface overheating or charge redistribution, causing partial desorption or reduced binding activity. Similar behavior has been reported in non-equilibrium systems under microwave exposure^39,40^. These findings are summarized in Fig. 5b. For the purpose of identifying the adsorption mechanism, the efficiency of removal was looked into kinetically.

During this investigation, four kinetic models were detected making use of second-order models (*PSO*) and pseudo-first order (*PFO*)^43^, Intraparticle, and Temkin models^18,44^.

The pseudo-first-order (PFO) model was initially applied as it represents adsorption controlled primarily by the availability of vacant sites on the adsorbent surface. The pseudo-first-order (PFO) model describes adsorption as a process mainly controlled by the number of vacant sites on the adsorbent surface. During the initial phase, the rapid adsorption of Pb^2+^ and MG suggests that numerous active sites are immediately available. According to this model, the amount adsorbed at any time t (qt, mg/g) is related to the equilibrium adsorption capacity (qe, mg/g) through a logarithmic relationship between ln (qe - qt) and time, as given in Eq. (3). The slope of this linear plot represents the rate constant K_1_ ​, indicating the speed at which the adsorption proceeds under the experimental conditions.

2 $${\text{ln }}\left( {{{\mathrm{q}}_{\mathrm{e}}} - {{\mathrm{q}}_{\mathrm{t}}}} \right){\text{ }}={\text{ ln}}{{\mathrm{q}}_{\mathrm{e}}} - {\text{ }}{{\mathrm{K}}_{\mathrm{1}}}{\mathrm{t}}~~~$$

The pseudo-second-order (PSO) model assumes that chemisorption governs the adsorption process, involving electron sharing or transfer between the adsorbate molecules and the functional groups on the nanocomposite surface. The high rate constant indicates a strong interaction between the pollutants and nanocomposite. In this study. This model is represented by 1/q_e_^2^ and t/q_t_ based upon Eq. (4), and following that, the rate constant, k_2_ (mg/g.sec), was derived as the reciprocal of the slope as indicated (4).

3 $${\mathrm{t}}/{{\mathrm{q}}_{\mathrm{t}}}={\text{ 1}}/{{\mathrm{k}}_{\mathrm{2}}}{{\mathrm{q}}_{\mathrm{e}}}^{{\mathrm{2}}}+{\text{ t/}}{{\mathrm{q}}_{\mathrm{e}}}~$$

The Intraparticle diffusion model describes the adsorption process as occurring in two main stages. In the first stage, the adsorbate ions (Pb^2+^ or MG) rapidly diffuse from the bulk solution toward the external surface of the nanocomposite. In the second stage, the ions gradually diffuse into the interior pores of the adsorbent. To evaluate whether Intraparticle diffusion is the sole rate-limiting step, a plot of qt versus t^1/2^ is constructed (Eq. 5). If the resulting line passes through the origin, it indicates that Intraparticle diffusion controls the adsorption rate exclusively. However, if the line does not pass through the origin, other mechanisms such as surface adsorption or external mass transfer also contribute to the overall rate. The slope of the linear portion of this plot K_id_ (mg.g^− 1^.sec^− 1/2^) is used to estimate the rate constant for intraparticle diffusion, providing insights into the relative contribution of pore diffusion to the overall adsorption process.

4 $${{\mathrm{q}}_{\mathrm{t}}}={\text{ }}{{\mathrm{k}}_{{\mathrm{id}}}}{{\mathrm{t}}^{{\mathrm{1}}/{\mathrm{2}}}}+{\text{ C}}$$

The Elovich model describes chemisorption on heterogeneous surfaces, where the adsorption rate decreases as surface coverage increases. The parameters α and β represent the initial adsorption rate and the effect of surface coverages demonstrated in Eq. (6).

5 $${{\mathrm{q}}_{\mathrm{t}}}={\text{ 1}}/\beta {\text{ ln}}\alpha \beta {\text{ }}+{\text{ 1}}/\beta {\text{ lnt}}$$

Whereas, the adsorption rate (mg/g.sec) is represented by α, and β is the Pb^2+^ or MG surface coverage beyond the bare minimum needed activation energy to initiate chemisorption as an adsorption technique.

The adsorption kinetics were analyzed using pseudo-first-order (PFO) and pseudo-second-order (PSO) models. The (PFO) model provided a better fit (R² > 0.98), indicating that chemisorption was likely the rate-limiting step. The high value of the PFO rate constant (k₂) suggests a strong affinity between Pb^2+^/MG and the active sites of the composite. This is consistent with electron-sharing or exchange mechanisms involving functional groups on the Bentonite@Perovskite@MOF surface. Similar kinetic behavior has been reported for comparable multifunctional nanocomposites^17,27,44^.

It is important to clarify that the Temkin model is an adsorption isotherm model, not a kinetic model, and its use in kinetic analysis is conceptually inaccurate. The Temkin model assumes that the heat of adsorption decreases linearly with coverage, which is relevant to equilibrium studies rather than adsorption rates. For a proper understanding of adsorption kinetics, models such as pseudo-first-order, pseudo-second-order, Elovich, and intra-particle diffusion should be applied. An in-depth analysis of the parameters obtained from these kinetic models can reveal the underlying adsorption mechanism, such as whether the process is governed by surface interactions, pore diffusion, or chemisorption. Recent studies utilizing advanced hydrogel nanocomposites and functionalized materials have shown that such kinetic assessments can differentiate between physical and chemical adsorption and identify rate-limiting steps^17,44^. A detailed evaluation of these models not only validates the adsorption efficiency of novel materials but also enhances mechanistic insight into their behavior in removing various contaminants from aqueous medium^23,24^.

#### Adsorption mechanism

Pb^2+^ and MG are mainly removed by electrostatic attractions and surface complexation mechanisms. Under suitable pH conditions, the nanocomposite surface acquires a negative charge, enhancing electrostatic interactions with positively charged Pb^2+^ ions and MG molecules. Furthermore, hydroxyl and metal–oxygen functional groups present in bentonite and perovskite Co–Ni oxides can coordinate with Pb^2+^ ions, indicating the involvement of chemisorption. In the case of MG, additional π–π interactions with the organic linkers of the MOF contribute to its adsorption. The mesoporous structure of the MOF also facilitates efficient diffusion of pollutants toward internal active sites, improving the overall removal performance.

#### Effect of Bent@Perov@BM-MOFs nanocomposite dosage

The influence of the Bent@Perov@BM-MOFs nanocomposite dosage on the removal efficiency of Pb^2+^ and MG was investigated using various adsorbent masses ranging from 10.0 to 100.0 mg (Fig. 5c). All experiments were conducted under optimal conditions: pH 7.0 for Pb^2+^ and pH 6.0 for MG contaminants, and contact times of 5 s for Pb^2+^ and 10 min for MG. For Pb^2+^, the adsorption capacity decreased significantly as the adsorbent dosage increased from 10.0 to 100.0 mg, with capacities recorded as 256.43, 88.50, 48.02, and 1.03 mg/g, respectively. A similar trend was observed for MG, with capacities of 14.04, 6.79, and 1.45 mg/g at 10.0, 20.0, and 100.0 mg doses, respectively. This inverse relationship between dosage and adsorption capacity per gram is attributed to the aggregation of adsorbent particles at higher doses, which leads to overlapping active sites and reduced available surface area per unit mass. Furthermore, when the adsorbent is present in excess relative to the contaminant concentration, the number of available binding sites surpasses the amount of pollutant molecules, resulting in underutilization of the surface. Similar findings have been reported for heavy metal and dye systems in nanocomposite-based adsorption studies.

A notable observation was the rapid uptake of Pb^2+^ within the first 5 s, followed by a slight decline. This uncommon behavior may be explained by surface saturation or partial desorption due to surface energy redistribution. The highly porous and reactive structure developed via microwave-assisted synthesis likely offers abundant binding sites at the beginning, which may subsequently undergo structural rearrangement or ion exchange. Such transient adsorption behavior is consistent with non-equilibrium systems dominated by fast kinetics^17,44^.

#### Screening experiments

To further evaluate the performance of the synthesized ternary composite, comparative adsorption experiments were carried out using the individual components (bentonite, Co–Ni perovskite, and Mg/Cu-MOFs) and their binary combinations. The ternary system demonstrated markedly superior adsorption capacities for both Pb^2+^ and MG compared to any single or binary system. This enhancement is attributed to synergistic effects, including increased surface area, diverse functional groups, and a higher density of active adsorption sites. These results align with recent reports emphasizing the importance of composite system optimization in environmental remediation^35,36,42,45^.

####  Effect of initial metal and dye concentration on adsorption

The effect of varying initial concentrations of Pb^2+^ (0.2, 0.4, 0.6, 0.8, 1, 1.2, 1.4, 1.6, 1.8,2 mol/L) and MG (10.0–100.0.0.0 mg/L) on adsorption performance was investigated (Fig. 5d). For both contaminants, an increase in initial concentration led to a notable increase in adsorption capacity. Conversely, when Pb^2+^ and MG concentrations were very low (i.e., trace levels), the removal efficiency was significantly reduced^27,38^. This can be attributed to mass transfer limitations between the solute and the active binding sites on the adsorbent surface. At low concentrations, the driving force for diffusion is weak, resulting in limited interaction between the adsorbate and the nanocomposite. The lowest removal value was 25.51, and 1.22 However, the most significant removal value was acquired by using1118.05 and 264.89, for Pb^2+^ and MG, respectively, finally this research is illustrated in Fig. 5d.

To determine the appropriate mechanism, the adsorption models of adsorption were examined in addition to the maximum adsorption onto the perovskite Ni-Co oxide @ Bimetallic MOFs [Mg/Cu-T] nanocomposite. In this study, the models of linear adsorption Freundlich and Langmuir were applied and looked into, and following that, the characteristics of adsorption were determined as listed in Table 5. The initial model of adsorption was Langmuir; The adsorption mechanism is regarded in this model as a unimolecular chemical reaction that has the ability to produce a reversible response. The adsorption in this model transpires on surface of the nanocomposite in a monolayer and is simultaneously homogeneous. Equation (7) pointed up the Langmuir model.

**Table 5:** Adsorption isotherm parameters for the adsorption of Pb^2+^ and MG onto Bent@Perov@BM-MOFs.

Adsorption model	Equations	Adsorption parameters	Pb^+ 2^	Malachite green
Langmuir	Ce/q_e_=1/q_max_ K_L_+ Ce/q_ma*x*_	q_m_ (mg g^− 1^)	45.45	1.78
		K_L_ (Lmg^− 1)^	0.025	3.025 E10^− 5^
		R^2^	0.963	0.839
Freundlich	Ln (qe) = Ln (K_F_) + 1/n Ln (C_e_)	n	1.88	1.29
		K_F_ (Lmg^− 1^)	2.92	0.13
		R^2^	0.960	0.798
Temkin	q_e_=(RT/b_T_) Ln(a_T_) + (RT/b_T_) ln (c_e_)	B (j/mol)	11.86	6.13
		a_T_ (Lg^− 1^)	− 1.53	− 3.78
		R^2^	0.981	0.919
Dubinin–Radushkevich (D–R)	Ln(q_e_) = Ln (q_s_) - (K_ad_ ἑ^2^)	q_s_ (mgg^− 1^)	15.30	35.48
		K_ad_ (mol^2^/Kj^2^)	−0.002	− 2.0 E10^− 5^
		R^2^	0.988	0.819

6 $${\mathrm{1}}/{{\mathrm{q}}_{\mathrm{e}}}={\text{ 1}}/{{\mathrm{q}}_{{\mathrm{max}}}}+{\mathrm{1}}/{{\mathrm{q}}_{{\mathrm{max}}}}{{\mathrm{K}}_{\mathrm{L}}}{{\mathrm{C}}_{\mathrm{e}}}$$

q_e_ (mg/g) is the equilibrium concentration of the adsorbates Pb^2+^ or MG on the adsorbent Bent@Perov@BM-MOFs nanocomposite. q_max_ (mg/g) is the greatest adsorption upon the surface of the nanocomposite. (Pb^2+^), and MG equilibrium concentration is Ce (mg/L^− 1^), where the saturation constant is depicted with KL (L mg^− 1^) as given in Table 5.

Freundlich was the second adsorption model. According to this framework, remediation should take accomplished via the adsorbates interaction with the adsorbent’s active binding sites on a heterogeneous surface, as stated by Eq. (8).

7 $${\mathrm{q}}_{\mathrm{e}}={\mathrm{K}}_{\mathrm{F}}{\mathrm{C}}_{\mathrm{e}}^{1/\mathrm{n}}$$

Another linear format of Eq. (8) is as described below;

8 $${\mathrm{ln}}{{\mathrm{q}}_{\mathrm{e}}}={\text{ ln }}{{\mathrm{K}}_{\mathrm{F}}}+{\text{ 1}}/{\text{n ln}}{{\mathrm{C}}_{\mathrm{e}}}$$

q_e_ is the adsorbed Pb^2+^, or MG (mg/g), C_e_ is the equilibrium of Pb^2+^, or MG in the liquid phase, Freundlich constant based on the energy of bonding is K_F_.

Based on the findings of this investigation, the conclusion reached was that Pb^2+^, or MG were fitted with Freundlich model. The specifications of the adsorption process were computed and shown in Table 5. The adsorption fitting with the Freundlich model establishes heterogeneous sites of the nanocomposite and the multilayer adsorption process^27,38,43^.

The Langmuir model assumes monolayer adsorption onto a surface with a finite number of identical sites. The high correlation coefficient (R² > 0.98) and the calculated maximum adsorption capacity (q_max_) in Table 6 indicate that Pb^2+^ and MG adsorption occur via monolayer coverage on homogeneous surfaces^27,38^.

**Table 6:** Non-linear regression analyses of isotherm and kinetics datasets.

Models and parameters	Pb^+ 2^	MG
Pseudo- second order kinetics	${\mathrm{q}}_{\mathrm{t}}=\frac{{{\mathrm{q}}_{\mathrm{e}}}^{2}{\mathrm{k}}_{2}\mathrm{t}}{1+{\mathrm{q}}_{\mathrm{e}}{\mathrm{k}}_{2}\mathrm{t}}$	
${\mathrm{q}}_{\mathrm{e}}(\mathrm{m}\mathrm{g}/\mathrm{g})$	43.47	14.49
${\mathrm{k}}_{2}(\mathrm{g}/\mathrm{m}\mathrm{g}.\mathrm{m}\mathrm{i}\mathrm{n}$)	145.52	32.02
${\mathrm{R}}^{2}$	0.772	0.992
Dubinin-Radushkevich (D-R) isotherm	${\mathrm{q}}_{\mathrm{e}}={\mathrm{q}}_{\mathrm{s}}{\mathrm{e}}^{-{\mathrm{k}}_{\mathrm{a}\mathrm{d}}{\varepsilon}^{2}}$ ${}^\varepsilon \varepsilon = {\mathrm{RT}}\ln \left( {1 + \frac{1}{{{{\mathrm{c}}_{\mathrm{e}}}}}} \right)$	
${\mathrm{q}}_{\mathrm{s}}(\mathrm{m}\mathrm{g}/\mathrm{g})$	15.30	35.48
${\mathrm{k}}_{\mathrm{a}\mathrm{d}}({\mathrm{m}\mathrm{o}\mathrm{l}}^{2}{\mathrm{k}\mathrm{J}}^{2}$	− 0.0.002	−2.0×${10}^{-5}$
${\mathrm{R}}^{2}$	O.988	0.819

The Freundlich model, which assumes multilayer adsorption on heterogeneous surfaces, also fit the data well. The Freundlich constant1/n was less than 1 for both contaminants, indicating favorable adsorption and surface heterogeneity^27,38^. The Dubinin–Radushkevich (D–R) model provided estimates of mean free energy (E) of adsorption. Values of E < 8 kJ/mol indicate a physisorption-dominant process, while higher values suggest ion exchange or surface complexation. In our case, the E values for Pb^2+^ were around X kJ/mol, indicating a possible chemisorption mechanism, while MG adsorption was primarily physisorptive^38,43^.

These findings suggest that both Pb^2+^ and MG follow mixed adsorption mechanisms involving electrostatic interaction, surface complexation, and ion exchange.

#### Interfering ions effect on the removal process

The interfering ions always affect the adsorption process according to the kind of nanocomposite and the kind of adsorbate. In this work, diverse interfering ions have been studied Na^+^, K^+^, NH_4_^+^, Mg^+ 2^, and Ca^+ 2^ using sodium chloride, potassium chloride, ammonium chloride, magnesium sulfate, and calcium sulfate, respectively. The removal capacity values have different behaviors according to the kind of interfering ions. In the case of the Pb^2+^ adsorption the Na^+^ interfering ion has the smallest decreasing effect on the adsorption process within an adsorption value of about 87.18 mg/g, conversely, highest effect on the adsorption was Mg^+ 2^ ions with within an adsorption value of about 62.43 mg/g, the order of decreasing on the adsorption values was as the following Mg^+ 2^< Ca^+ 2^< NH_4_^+^ < K^+^< Na^+^. In the case of the MG adsorption in existence of the K^+^ interfering ion has the smallest decreasing effect on the adsorption process within an adsorption value of about 14.14 mg/g, conversely, the greatest effect on the adsorption was Ca^+ 2^ ions with within an adsorption value of about 10.10 mg/g, the order of decreasing on the adsorption values was as the following Ca^+ 2^< Mg^+ 2^< NH_4_^+^ < Na^+^< K^+^. Finally, the he calculated adsorption capacity is presented in Fig. 5e, where it is compared with the results obtained under non-interfering conditions^27,43,46^.

#### Implementation of the Bent@Perov@BM-MOFs in a micro-column

The final step of this work was the testing of Bent@Perov@BM-MOFs nano sorbent to remediate Pb^2+^ and MG by means of the microcolumn technology Bent@Perov@BM-MOFs nanocomposite was employed to fix 10.0 mg L of Pb^2+^ or MG was spiked in tap water, and industrial waste water. A multi-stage microcolumn system containing 10.0 mg of the nanocomposite was used for this procedure. were distributed above a glass wall above in the bottom of the microcolumn end. The obtained results were stated in Table 7. The values that are removed and recovered Pb^2+^ and MG from water and wastewater using the synthesized nano sorbent demonstrated good resemblances. Following the third run, the mean proportion of adsorption from water was demonstrated, and were found as 92.0% and 94.50% for Pb^2+^ and MG, respectively, while for waste water were 90.0% and 89.50%, respectively to ascertain the reliability of this nano sorbent to remediate Pb^2+^and MG from tap water and wastewater.

**Table 7:** Remediation of Pb^2+^ and MG from tap water and real industrial wastewater.

Cycle	Pb^2+^ (tap water)	Pb^2+^ (wastewater)	MG (tap water)	MG (wastewater)
1	92.0%	90.30%	95.0%	91.50%
2	94.5%	88.60%	92.5%	90.20%
3	90.0%	87.20%	91.0%	88.50%
4	88.30%	85.90%	89.60%	86.80%
5	86.70%	85.0%	88.20%	85.10%

#### Leaching and stability of Co and Ni Ions

To assess the environmental safety of the Bent@Perov@BM-MOFs nanocomposite, leaching studies were conducted under both neutral, and acidic conditions. After adsorption tests at pH 7.0 and pH 3.0, the concentration of cobalt and nickel ions released into the solution was measured using ICP-OES. The leached concentrations of Co^2+^ and Ni^2+^ were found to be 0.032 mg/L and 0.029 mg/L at pH 7.0, and 0.044 mg/L and 0.041 mg/L at pH 4.0, respectively. These values are significantly below the WHO and EPA permissible limits for drinking water, indicating minimal risk of secondary pollution and confirming the structural stability of the composite.

#### Reusability and regeneration study

To assess the regeneration capability and reusability of the synthesized composite, adsorption–desorption experiments were conducted over five consecutive cycles. After each adsorption cycle, the used material was rinsed with ethanol and distilled water and then dried at 60.0 °C before reuse. Bent@Perov@BM-MOFs maintained over 85.0% removal efficiency for both Pb^2+^ and MG after five cycles, demonstrating excellent stability and reusability. These results highlight the material’s potential for repeated use in practical water purification applications.

## Conclusion

**Fig. 6:**
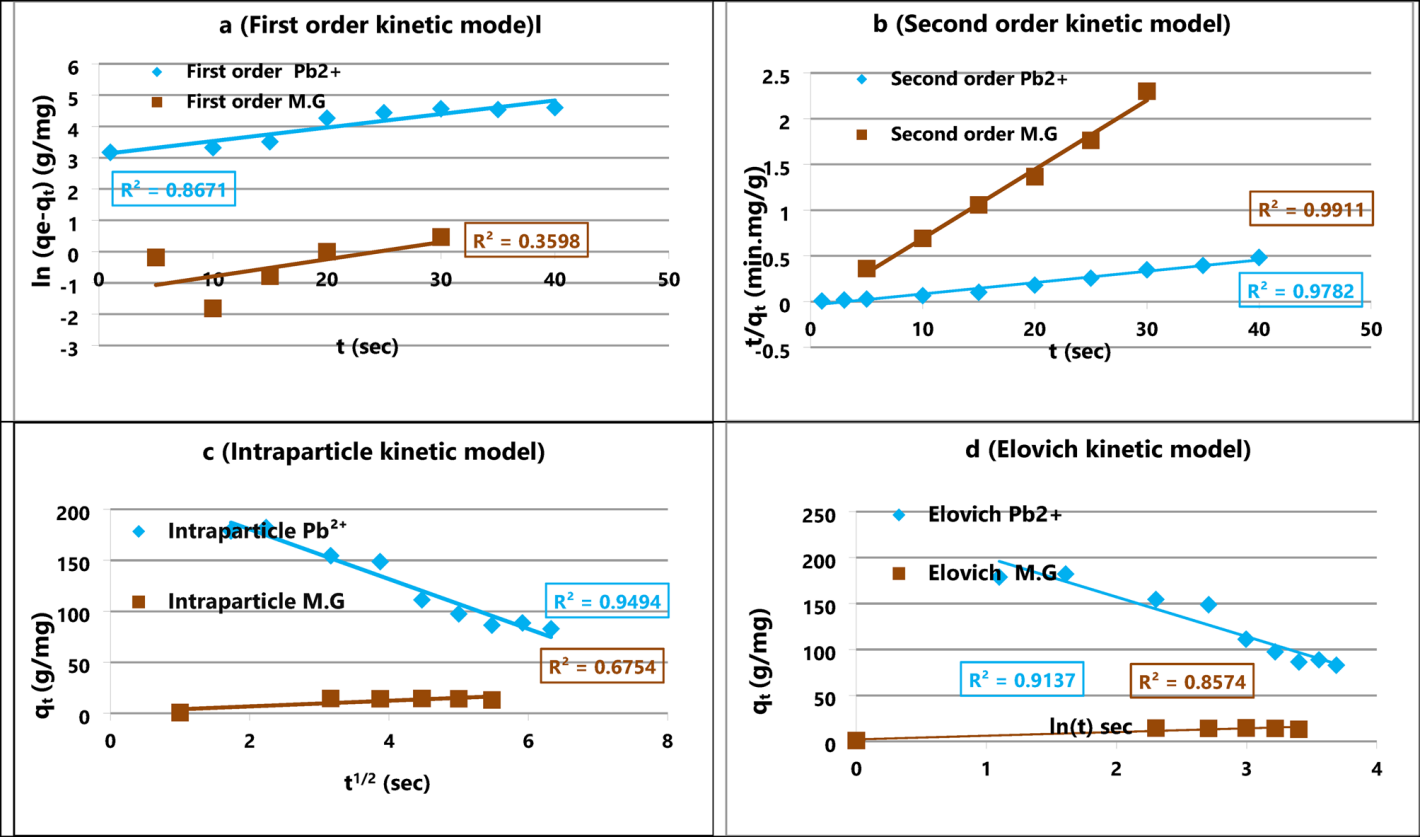
(**a**–**d**) Kinetics f Pb^2+^ and malachite green onto Bent@Perov@BM-MOFs nanosorbent.

The assembled nanocomposite material in this work was composed of intercalated bentonite with Ni–Co perovskite oxide and Bimetallic Mg/Cu–tartaric acid MOFs, was synthesized via a green approach and characterized for its efficiency in removing Pb^2+^ and MG from aqueous solutions (Fig. 6). The Bimetallic Mg/Cu–tartaric MOFs were prepared using a co-precipitation method, and the composite was characterized using FT-IR, XRD, BET surface area determination, TGA, and SEM techniques to evaluate its chemical and morphological properties. The nanocomposite was tested for its adsorption performance toward Pb^2+^ and MG. The maximum adsorption capacity for Pb^2+^ was found to be 105.70 mg/g at pH 7.0 using 10.0 mg of the adsorbent within just 5.0 s. For MG, the optimal conditions were pH 6.0, dye concentration of 20 mg/L, a contact time of 20.0 min, and 10.0 mg of the adsorbent, achieving a maximum capacity of 14.53 mg/g. The adsorption kinetics for both contaminants conformed to a pseudo-second-order model. Isotherm analysis demonstrated that Pb^2+^ adsorption best fit the Dubinin–Radushkevich (D–R) model, while MG followed the Temkin isotherm model. The estimated material cost for synthesizing 1.0 g of Bent@Perov@Bi-MOFs nanocomposite (Lab Scale) is listed out in Table 8. These findings confirm the potential of the synthesized nanocomposite as an efficient and rapid adsorbent for environmental remediation of toxic metal ions and organic dyes compared to previously reported materials as listed in Table 9.

**Table 8:** Estimated material cost for synthesizing 1.0 g of Bent@Perov@Bi-MOFs nanocomposite (Lab Scale).

Component	Chemical	Amount used	Approx. unit price (USD/g)	Estimated cost (USD)
Clay base	Bentonite powder	1.0 g	0.02	0.02
Cobalt nitrate hexahydrate	Co (NO₃)₂·6 H₂O	0.2 g	0.12	0.024
Nickel nitrate hexahydrate	Ni (NO₃) ₂·6 H₂O	0.2 g	0.10	0.020
Magnesium nitrate hexahydrate	Mg (NO₃) ₂·6 H₂O	0.2 g	0.08	0.016
Cupper nitrate trihydrate	Cu (NO₃) ₂·3 H₂O	0.2 g	0.09	0.018
Organic linker	Tartaric acid	0.3 g	0.05	0.015
Solvent	Deionized water (~ 100 mL)	—	Negligible	0.000
Energy & processing	Heating, drying, etc.	—	Estimated	0.10
Miscellaneous	Glassware, minor reagents	—	Estimated	0.05

**Table 9:** Comparison of adsorption performance of the synthesized composite with reported adsorbents for Pb^2+^ and malachite green.

Adsorbent	Target	q_max_ (mg/g)	pH	Contact Time (min)	Reference
Bentonite-based MOF	Pb^2+^	105.70	7.0	0.08 min )5.0 s(	This study
Activated carbon	Pb^2+^	89.50	5.5	60.0 min	^20^
Chitosan–Fe₃O₄ nanocomposite	Pb^2+^	112.30	6.0	30.0 min	^19^
Clay–ZnO composite	MG	12.40	6.5	30.0 min	^29^
MOF-5 composite	MG	14.10	6.0	20.0 min	^33^
Natural zeolite	Pb^2+^ and MG	40–70 for Pb^2+^ and 30–60 for MG	5–7	60–120 min	^45^
CuS-NPs/AC	Pb^2+^ and MG	90–120 for Pb^2+^ and 70–100 for MG	6–7	90 min	^46^
Clay-based composite	Pb^2+^ and MG	60–110 for Pb^2+^ and 50–90 for MG	6–8	120 min	^38^
Current study composite	MG	14.53	6.0	20.0 min	This study

## Supplementary Information

Below is the link to the electronic supplementary material.

Supplementary Material 1

## Data Availability

The datasets generated and analyzed during the current study are available from the corresponding author on responsible request.

## References

[1] Hayat, A. et al. Recent advance in MOFs and MOF-based composites: Synthesis, properties, and applications. *Mater. Today Energy*10.1016/j.mtener.2024.101542 (2024).

[2] Thakur, S. & Bharti, S. Unlocking the potential of metal–organic frameworks: A review on synthesis, characterization, and multifaceted applications. *J. Inorg. Organomet. Polym. Mater.* **34**(10), 4477–4508 (2024).

[3] Huang, D. et al. Preparation of metal–organic frameworks with bimetallic linkers and corresponding properties. *New J. Chem.* **43** (19), 7243–7250 (2019).

[4] Zhang, Z., Liu, J., Wang, Z. & Zhang, J. Bimetallic Fe–Cu-based metal–organic frameworks as efficient adsorbents for gaseous elemental mercury removal. *Ind. Eng. Chem. Res.* **60** (1), 781–789 (2020).

[5] Wang, J. et al. MOFs derived Co/Cu Bimetallic nanoparticles embedded in graphitized carbon nanocubes as efficient Fenton catalysts. *J. Hazard. Mater.* **394**, 122567 (2020). 32229387 10.1016/j.jhazmat.2020.122567

[6] Ma, J., Liu, T., Ye, W., He, Q. & Chen, K. High-entropy perovskite oxides for energy materials: A review. *J. Energy Storage*. **90**, 111890 (2024).

[7] Lin, N., Gong, Y., Wang, R., Wang, Y. & Zhang, X. Critical review of perovskite-based materials in advanced oxidation system for wastewater treatment: Design, applications and mechanisms. *J. Hazard. Mater.* **424**, 127637 (2022). 34753649 10.1016/j.jhazmat.2021.127637

[8] Allam, E. A., Ghamry, M. A., Gizawy, M. A., El-Sharkawy, R. M. & Mahmoud, M. E. Investigation of adsorption optimization, kinetic and isotherm behaviors of 60Co and 152 + 154Eu radioisotopes from nuclear radioactive wastewater onto a novel Co0. 5Ni0. 5O–Co2Mo3O8–CuO–ZnO perovskite metal oxides nanosorbent. *J. Inorg. Organomet. Polym Mater.* **34** (11), 5551–5565 (2024).

[9] Hassanin, M. A. et al. Uptake of uranium (VI) upon a novel developed nanocomposite of bimetallic Mg/Mn bentonite lupine peels. *J. Water Process. Eng.* **66**, 106009 (2024).

[10] Sherin, A. et al. Reactive Blue MEBF 222 dye and textile wastewater treatment using metal-doped cobalt and nickel perovskites by batch and column adsorption process. *Environ. Monit. Assess.* **196** (10), 927 (2024). 39266805 10.1007/s10661-024-13035-w

[11] Zhao, X. et al. Multifunctional bacterial cellulose-bentonite@polyethylenimine composite membranes for enhanced water treatment: Sustainable dyes and metal ions adsorption and antibacterial properties. *J. Hazard. Mater.* **477**, 135267 (2024). 39047552 10.1016/j.jhazmat.2024.135267

[12] EL Kaim Billah, R. et al. Methyl Orange adsorption studies on glutaraldehyde cross-linking chitosan/fluorapatite-based natural phosphate composite. *Int. J. Environ. Anal. Chem.* **104**(17), 5840–5856 (2024).

[13] Aziz, K. H. H. et al. Heavy metal pollution in the aquatic environment: Efficient and low-cost removal approaches to eliminate their toxicity: A review. *RSC Adv.* **13**(26), 17595–17610 (2023). 37312989 10.1039/d3ra00723e PMC10258679

[14] Zhang, Y., Wang, Z., Liu, X., Chen, X. & Li, Y. In situ synthesis of Pd‑doped ZnO with enhanced visible‑light photocatalytic activity for the degradation of sulfamethoxazole. *Chem. Eng. J.* **440**, 140446 (2022).

[15] Khan, T. A., Rahman, R. & Equbal, E. A. Decolorization of Bismarck Brown R and Crystal Violet in liquid phase using modified pea peels: Non-linear isotherm and kinetics modeling. *Model. Earth Syst. Environ.* **2**(3), 141 (2016).

[16] Singh, B. & Gupta, H. Metal–organic frameworks (MOFs) for hybrid water electrolysis: Structure–property–performance correlation. *Chem. Commun.* **60**(62), 8020–8038 (2024). 10.1039/d4cc02729a 38994743

[17] Khan, S. A., Siddiqui, M. F. & Khan, T. A. Ultrasonic-assisted synthesis of polyacrylamide/bentonite hydrogel nanocomposite for the sequestration of lead and cadmium from aqueous phase: Equilibrium, kinetics and thermodynamic studies. *Ultrason. Sonochem.* **60**, 104761 (2020). 31499323 10.1016/j.ultsonch.2019.104761

[18] Aljeboree, A. M., Alshirifi, A. N. & Alkaim, A. F. Thermodynamic and kinetic study of adsorption of heavy metals on new synthesized adsorbents. *Arab. J. Chem.* **15**(7), 103925 (2022).

[19] Li, Y., Liu, H., Wang, W. & Jin, L. Efficient removal of heavy metals from aqueous solution by a bioadsorbent derived from modified chitosan. *Int. J. Biol. Macromol.* **134**, 390–400 (2019).31078599

[20] Khazri, H., Ghorbel-Abid, I., Kalfat, R. & Trabelsi-Ayadi, M. Lead removal from aqueous solution by natural and modified bentonite: Adsorption performance and mechanisms. *Environ. Res.* **175**, 76–85 (2019).

[21] Levallois, P., Barn, P., Valcke, M., Gauvin, D. & Kosatsky, T. Public health consequences of lead in drinking water. *Curr. Environ. Health Rep.* **5**, 255–262 (2018). 29556976 10.1007/s40572-018-0193-0

[22] Bhatia, M., Babu, S. & Sonawane, R. Application of nanoadsorbents for removal of lead from water. *Int. J. Environ. Sci. Technol.* **14**, 1135–1154 (2017).

[23] Mahmoud, M. E., Adel, S. E. & ElSayed, I. E. T. Development of titanium oxide-bound-α-aminophosphonate nanocomposite for adsorptive removal of lead and copper from aqueous solution. *Water Resour. Ind.* **23**, 100126 (2020).

[24] Luo, X. et al. Adsorptive removal of lead from water by the effective and reusable magnetic cellulose nanocomposite beads entrapping activated bentonite. *Carbohydr. Polym.* **151**, 640–648 (2016). 27474609 10.1016/j.carbpol.2016.06.003

[25] Geisse, A. R., Ngule, C. M. & Genna, D. T. Removal of lead ions from water using thiophene-functionalized metal–organic frameworks. *Chem. Commun.* **56** (2), 237–240 (2020). 10.1039/c9cc09022c 31803866

[26] Li, Y. et al. Removal of lead (Pb^2+^) from contaminated water using a novel MoO_3_-biochar composite: Performance and mechanism. *Environ. Pollut.* **308**, 119693 (2022). 35777593 10.1016/j.envpol.2022.119693

[27] Shi, Z. et al. Magnetic metal organic frameworks (MOFs) composite for removal of lead and malachite green in wastewater. *Colloids. Surf. A. Physicochem. Eng. Aspects.* **539**, 382–390 (2018).

[28] Wang, H., Chen, Q., Liu, R., Zhang, Y. & Zhang, Y. Synthesis and application of starch-stabilized Fe–Mn/biochar composites for the removal of lead from water and soil. *Chemosphere* **305**, 135494 (2022). 35764108 10.1016/j.chemosphere.2022.135494

[29] Altıntıg, E. et al. Facile synthesis of zinc oxide nanoparticles loaded activated carbon as an eco-friendly adsorbent for ultra-removal of malachite green from water. *Environ. Technol. Innov.* **21**, 101305 (2021).

[30] Khawaja, H., Zahir, E., Asghar, M. A. & Asghar, M. A. Graphene oxide decorated with cellulose and copper nanoparticle as an efficient adsorbent for the removal of malachite green. *Int. J. Biol. Macromol.* **167**, 23–34 (2021). 33259838 10.1016/j.ijbiomac.2020.11.137

[31] Amiri, M., Salavati-Niasari, M., Akbari, A. & Gholami, T. Removal of malachite green (a toxic dye) from water by cobalt ferrite silica magnetic nanocomposite: Herbal and green sol-gel autocombustion synthesis. *Int. J. Hydrog. Energy.* **42**(39), 24846–24860 (2017).

[32] Wang, D., Liu, L., Jiang, X., Yu, J. & Chen, X. Adsorption and removal of malachite green from aqueous solution using magnetic β-cyclodextrin-graphene oxide nanocomposites as adsorbents. *Colloids. Surf. A. Physicochem. Eng. Aspects.* **466**, 166–173 (2015).

[33] Shi, Z. et al. Renewable metal–organic-frameworks-coated 3D printing film for removal of malachite green. *RSC Adv.* **7** (79), 49947–49952 (2017).

[34] Murthy, T. K., Gowrishankar, B. S., Prabha, M. C., Kruthi, M. & Krishna, R. H. Studies on batch adsorptive removal of malachite green from synthetic wastewater using acid treated coffee husk: Equilibrium, kinetics and thermodynamic studies. *Microchem. J.* **146**, 192–201 (2019).

[35] Khan, S. A., Abbasi, N., Hussain, D. & Khan, T. A. Sustainable mitigation of paracetamol with a novel dual-functionalized pullulan/kaolin hydrogel nanocomposite from simulated wastewater. *Langmuir* **38** (27), 8280–8295 (2022). 35758902 10.1021/acs.langmuir.2c00702

[36] Zha, X., Zhao, X., Webb, E., Khan, S. U. & Wang, Y. Beyond pristine metal–organic frameworks: Preparation of hollow MOFs and their composites for catalysis, sensing, and adsorption removal applications. *Molecules* **28**(1), 144 (2022). 36615337 10.3390/molecules28010144 PMC9821992

[37] Hussain, D., Khan, S. A. & Khan, T. A. Fabrication and characterization of mesoporous guar gum/NiWO_4_ nanocomposite for efficient adsorption of phloxine B and crystal violet from aqueous solution and evaluation of its antioxidant activity. *Colloid Interface Sci. Commun.* **44**, 100488 (2021).

[38] Du, B. et al. A novel modified lignin-based adsorbent for removal of malachite green and Pb^2+^ ions from wastewater. *Sep. Purif. Technol.* **330**, 125495 (2024).

[39] Khan, T. A., Fuzail Siddiqui, M., Abbasi, N. & Alharthi, S. S. Adsorptive decolouration of anionic dye from water by goat dropping activated carbon prepared via microwave-assisted H_3_PO_4_ activation: Process optimization using response surface methodology, isotherm and kinetics modelling. *Biomass Convers. Biorefinery* **12**(11), 5409–5425 (2022).

[40] Abbasi, N., Khan, S. A. & Khan, T. A. Statistically optimised sequestration of mefenamic acid from polluted water by acacia gum phthalate/pectin hydrogel: A novel multifunctional adsorbent material synthesised via microwave-assisted process. *Chem. Eng. J.* **466**, 143296 (2023).

[41] Srivastava, S., Sinha, R. & Roy, D. Toxicological effects of malachite green. *Aquat. Toxicol.* **66**(3), 319–329 (2004). 15129773 10.1016/j.aquatox.2003.09.008

[42] Bambal, A., Gaydhane, A., Chute, A., Sarvanan, D. & Jugade, R. Novel chitosan-magnetite-silica ternary capsules for highly efficient sequestration of reactive dyes from aqueous media. *Environ. Res.* **275**, 121359 (2025). 40086572 10.1016/j.envres.2025.121359

[43] Li, J., Liu, Y., Wang, D. & Chen, W. Insight into adsorption kinetics and mechanisms of pollutants onto engineered materials. *Chemosphere* **289**, 133232 (2022).34896178

[44] Singh, R., Kumar, A., Sharma, P. & Gupta, R. K. Kinetic and mechanistic evaluation of advanced adsorbents for wastewater remediation. *Langmuir* **38**(27), 8280–8295 (2022).35758902

[45] Wang, S. & Ariyanto, E. Competitive adsorption of malachite green and Pb^2+^ on adsorption natural zeolite. *J. Colloid Interface Sci.* **325**, 504–511 (2008). 10.1016/j.jcis.2007.05.032 17543322

[46] Fan, Z. et al. Competitive adsorption of malachite green and Pb^2+^ ions by CuS nanoparticles on activated carbon. *J. Environ. Chem. Eng.* **6**(3), 3456–3465 (2018).

