# Supplementary material for: Adsorption of Pb2+and malachite green from water onto a newly developed nanocomposite of bentonite@perovskite Co-Ni oxide@bimetallic Mg/Cu MOFs and their adsorption and kinetic studies
Source: Sci Rep. 2026 Apr 27;16:13520. doi: 10.1038/s41598-026-42785-5 (PMC13121628; doi:10.1038/s41598-026-42785-5)
Supplement: Supplementary file 1 — Supplementary Material 1 [file 41598_2026_42785_MOESM1_ESM.docx]

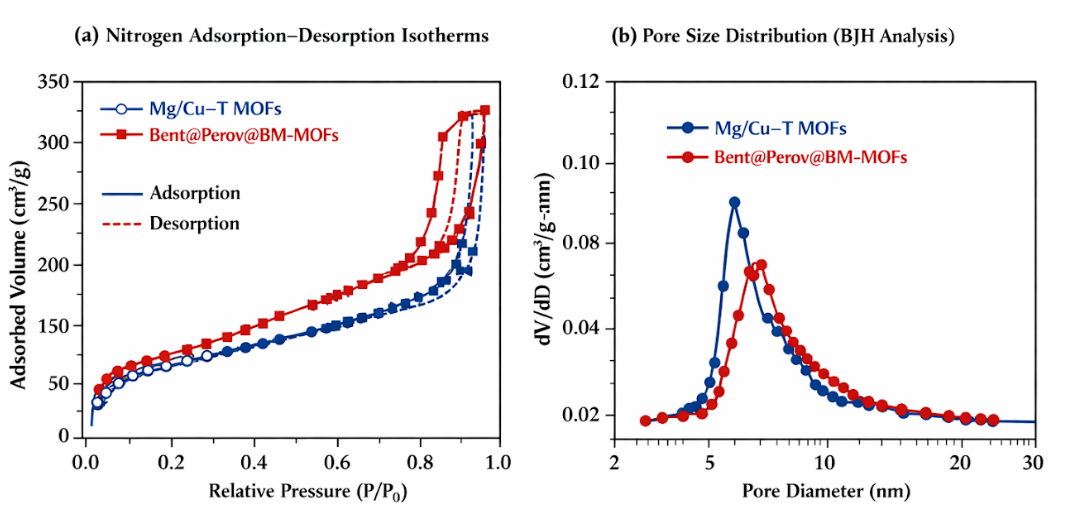


**Fig 1S.** (a) Nitrogen Adsorption-Desorption Isotherms of Mg/Cu-T MOFs and Bent@Perov@BM-MOFs; (b) Pore Size Distribution (BJH Analysis) of Mg/Cu-T MOFs and Bent@Perov@BM-MOFs.
